# Machine learning applied to fMRI patterns of brain activation in response to mutilation pictures predicts PTSD symptoms

**DOI:** 10.1186/s12888-023-05220-x

**Published:** 2023-10-05

**Authors:** Liana Catarina Lima Portugal, Taiane Coelho Ramos, Orlando Fernandes, Aline Furtado Bastos, Bruna Campos, Mauro Vitor Mendlowicz, Mariana da Luz, Carla Portella, William Berger, Eliane Volchan, Isabel Antunes David, Fátima Erthal, Mirtes Garcia Pereira, Leticia de Oliveira

**Affiliations:** 1 Neurophysiology Laboratory, Department of Physiological Sciences, Roberto Alcantara Gomes Biology Institute, Biomedical Center, Universidade do Estado do Rio de Janeiro, Boulevard 28 de Setembro, 87 - Vila Isabel, Rio de Janeiro, RJ 20551-030 Brazil; 2 Laboratory of Neurophysiology of Behavior, Department of Physiology and Pharmacology, Biomedical Institute, Universidade Federal Fluminense, R. Prof. Hernani Pires de Mello, 101, São Domingos, Niterói, RJ 24210-130 Brazil; 3 Mídiacom Lab, Institute of Computing, Universidade Federal Fluminense, Av. Gal. Milton Tavares de Souza, s/n, São Domingos, Niterói, RJ 24210-310 Brazil; 4 Laboratório de Neurobiologia, Instituto de Biofísica Carlos Chagas Filho, Universidade Federal do Rio de Janeiro, 373 - Cidade Universitária da Universidade Federal do Rio de Janeiro, Rio de Janeiro, RJ 21941-902 Brazil; 5 Linpes, Institute of Psychiatry, Universidade Federal do Rio de Janeiro, Av. Venceslau Brás, 71 - Botafogo, Rio de Janeiro, RJ 22290-140 Brazil

**Keywords:** PTSD, Machine learning, fMRI

## Abstract

**Background:**

The present study aimed to apply multivariate pattern recognition methods to predict posttraumatic stress symptoms from whole-brain activation patterns during two contexts where the aversiveness of unpleasant pictures was manipulated by the presence or absence of safety cues.

**Methods:**

Trauma-exposed participants were presented with neutral and mutilation pictures during functional magnetic resonance imaging (fMRI) collection. Before the presentation of pictures, a text informed the subjects that the pictures were fictitious (“safe context”) or real-life scenes (“real context”). We trained machine learning regression models (Gaussian process regression (GPR)) to predict PTSD symptoms in real and safe contexts.

**Results:**

The GPR model could predict PTSD symptoms from brain responses to mutilation pictures in the real context but not in the safe context. The brain regions with the highest contribution to the model were the occipito-parietal regions, including the superior parietal gyrus, inferior parietal gyrus, and supramarginal gyrus. Additional analysis showed that GPR regression models accurately predicted clusters of PTSD symptoms, nominal intrusion, avoidance, and alterations in cognition. As expected, we obtained very similar results as those obtained in a model predicting PTSD total symptoms.

**Conclusion:**

This study is the first to show that machine learning applied to fMRI data collected in an aversive context can predict not only PTSD total symptoms but also clusters of PTSD symptoms in a more aversive context. Furthermore, this approach was able to identify potential biomarkers for PTSD, especially in occipitoparietal regions.

**Supplementary Information:**

The online version contains supplementary material available at 10.1186/s12888-023-05220-x.

## Background

Posttraumatic stress disorder (PTSD) is an incapacitating psychiatric condition that some individuals may develop after exposure to traumatic events. Before the COVID-19 pandemic, the cross-national lifetime prevalence of PTSD was 3.9% [1]. Recently, in the current pandemic, millions of people worldwide have experienced symptoms of PTSD [2–7]. According to the Diagnostic and Statistical Manual of Mental Disorders 5th Edition (DSM-5, [8]), PTSD is diagnosed based on clusters of symptoms, such as re-experiencing the trauma, intrusive memories (criterion B), avoidance (criterion C), negative cognition and mood (criterion D), and hyperarousal (criterion E).

The categorical view of mental disorders provided by the DSM-5 and the International Classification of Diseases-11th version (ICD-11), offers benefits for clinical practice, such as reliability and an easier way of diagnosing psychiatric disorders across various contexts. However, mental health disorders are heterogeneous regarding symptom presentation, disease course, comorbidity, and biological underpinnings which can difficult the search for PTSD biomarkers. Thus, the National Institute of Mental Health (NIMH) developed the Research Domain Criteria (RDoC) to advocate a dimensional approach to understanding the pathophysiological processes underlying mental health disorders. A significant contribution was the conceptualization of psychopathology as encompassing multiple domains with integrative functions that span the full range of human behavior from normal to abnormal [9, 10].

Over the last fifteen years, machine learning approaches have been increasingly used to identify multivariate patterns in neuroimaging data that are predictive of diagnosis (pattern classification) or symptoms (pattern regression) at the individual subject level (for reviews, see [11–19]. One encouraging tool for identifying objective psychiatric illness markers is pattern recognition. In pattern recognition, the model captures an association between patterns and labels, allowing it to retrieve this association in samples it has never seen before. To develop pattern recognition models, the dataset is typically split into training and test sets. The training set is used to construct and train the model, while the test set is used to evaluate its performance. In brain studies, we can apply a regression model to identify brain signatures on functional magnetic resonance imaging (fMRI) data predictive of psychopathological symptoms [16–18]. This method may be more sensitive to spatially distributed effects in the brain than a standard mass-univariate analysis. Thus, it potentially provides an approach with higher detection power, for studies on subclinical populations with mild alterations [20, 21].

Few neuroimaging studies have applied pattern regression to predict clinical scores of PTSD at an individual level. The existing studies have been based only on resting state fMRI (rs-fMRI) data, which are often used for biomarker discovery due to their less constrained nature [22–25]. To date, no study has applied pattern regression analysis to find brain signatures from data collected while participants perform specific tasks related to PTSD psychopathology. To expand upon findings obtained from resting-state fMRI data, we used a paradigm in which two important contexts for trauma research were evaluated: engagement in safety cues and negative emotional reactivity [26–29]. For example, it is well established in the literature that patients with PTSD exhibit heightened processing of negative stimuli [26]. Additionally, patients with PTSD consistently show persistent and exaggerated threat responses in conditioning research paradigms even in the presence of safety cues [27–29]. Specifically, individuals without PTSD exhibited attenuated fear responses in the presence of a safety cue; however, patients with PTSD were unable to modulate their fear response in the presence of safety cues. The authors argued that the inability to engage in safety cues and respond to adaptive behaviors is a relevant biomarker of PTSD.

Instead of using a conditioning paradigm, we used more implicit safety cues, as this approach allows the investigation of the brain network in a more realistic context; it does not have the inconveniences of using a painful aversive stimulus (as in the conditional paradigm) and no “training” is needed. Here, we adopted an experimental approach in which participants were exposed to pictures of mutilated and neutral human body parts in “safe” and “real” contexts. At the beginning of each experimental run, a text informed participants that the pictures to be presented were the work of a make-up artist (“safe context”) or scenes from a journalistic report (“real context”). However, all pictures used were real scenes. In fact, previous studies by our group have shown an association between electroencephalography (EEG) responses to mutilation pictures and PTSD symptoms in a trauma-exposed cohort in a real context indicating that PTSD symptoms may be associated with increased negative emotional reactivity [30, 31]. Furthermore, when mutilation pictures were presented in a safe context, nonclinical participants showed attenuated autonomic [32, 33] and brain responses [34–36]. However, this attenuation of brain reactivity was not observed in patients with PTSD, indicating an impairment in engaging safety cues [36]. The present study aimed to apply multivariate pattern recognition methods to predict PTSD symptoms from whole-brain activation patterns in two contexts where the aversiveness of unpleasant pictures was manipulated by the presence or absence of safety cues. Based on previous results, we expect that this approach will identify brain activity patterns that can be used to predict PTSD symptoms at the individual level in two important contexts for trauma research: negative emotional reactivity (real context) and engagement in safety cues during exposure to negative pictures (safe context). Specifically, in the real context, our hypothesis was that machine learning algorithms would capture the relationship between the variations in the reactivity to negative stimuli in patterns of brain activation and PTSD symptoms. In the safe context, we expected that machine learning would capture the variability in emotional response attenuation and its association with PTSD symptoms. For the significant models, we also sought to determine, in an exploratory purpose analysis, whether patterns of brain activity could predict clusters of PTSD symptoms (e.g., intrusion, avoidance, negative alteration in cognition and mood and hyperarousal) derived from participant-reported scales scores. The adopted strategy can potentially identify brain biomarkers for PTSD.

## Methods

In this section, we describe the methods used in our analysis. We analyzed a sample of participants exposed to traumatic events with different levels of PTSD symptoms (with or without a PTSD diagnosis). Participants were exposed to images of body parts neutral and mutilated body parts. These images were presented in a real context (no safety cue) and in a safe context with a safety cue suggesting that images were fake. We collected fMRI data from the patients while observing the images in both contexts. We used a general linear model analysis to obtain the beta coefficient of the association of each voxel with the type of image (mutilated or neutral) and context (real or safe). Then, we trained a machine learning regression model to predict each patient’s PTSD Checklist for the DSM-5 (PCL-5) scores (PTSD symptoms) from its voxel beta coefficients. The machine learning regression model learns the association pattern in the training set, and we evaluate the model’s performance in the validation set using a cross-validation approach.

### Sample

To investigate whether PTSD symptoms could be decoded from whole-brain activity in response to real and safe contexts, we reanalyzed a dataset from Bastos et al. [36]. Our final sample included 43 participants: 20 with PTSD (15 women; mean age 29.0 ± 12.70 years) and 23 trauma-exposed participants without PTSD (15 women; mean age 34.4 ± 11.54 years). Both groups were assessed with the Structures Clinical Interview for the DSM-IV (SCID-IV) performed by experienced psychiatrists. For detailed characteristics of the participants and traumatic events, see Bastos et al. [36], and the Supplementary Material (Table S1). For information on data preprocessing procedures, see the section Data preprocessing section in the Supplementary Material.

This study was carried out in accordance with the tenets of the Declaration of Helsinki. It was approved by the Ethics Review Board of the Universidade Federal do Rio de Janeiro (process number 1.749.604). No participants were aware of the purpose of the experiment, and all signed informed consent forms before the assessment. Additionally, participants were informed of their right to withdraw from the study at any time.

### Symptom assessment

PTSD symptoms were assessed using the Posttraumatic Stress Disorder Checklist 5 (PCL-5, (American Psychiatric Association [APA], [37]). This scale was translated and adapted to Portuguese by Lima et al. [38]. The participants were instructed to complete the PCL-5 considering their worst traumatic experience reported on the Life Events Checklist 5 (LEC-5). The LEC-5 [38, 39] is an instrument that assesses exposure to traumatic events meeting diagnostic criterion A for PTSD. The PCL-5 is a 20-item self-report questionnaire that measures four clusters of symptoms of PTSD: intrusion (criterion B), avoidance (criterion C), negative alterations in cognition and mood (criterion D), and alterations in arousal and reactivity (criterion E). Each item on the PCL-5 questionnaire is assessed on a five-point Likert scale (from 0 = “not at all” to 4 = “extremely”). Total symptom severity can be calculated by summing the scores on all 20 items; the severity score ranges from zero to 80 points. The symptom clusters of PTSD can be calculated by totalling the items for each cluster separately: intrusion (min = 0, max = 20), avoidance (min = 0, max = 8), negative alterations in cognition and mood (min = 0, max = 28), and arousal/reactivity (min = 0, max = 24).

### Experimental design

The experiment involved two sets of pictures: the neutral set (n = 60) and the mutilation set (n = 60). The neutral set included pictures of body parts in typical life situations. The mutilation set included pictures of real scenes of human body parts with lacerations. Both sets were composed of images selected from a dataset of real images. As previously described by Bastos et al. [36], the images were presented in two different contexts: the “real” context and “safe” context. The real context was defined by the presentation of text instructing the participant that the pictures to be presented were from journalistic material (real scenes). The safe context was defined by the presentation of a text instructing the participants that the pictures to be presented were the work of a makeup artist (not real scenes). The text aimed to modulate the perceived aversiveness for each set of images. The pictures presented in the safe context for half of the subjects were presented in the real context for the other half.

Each context comprised two runs. In each run, the participant was presented with 15 pictures of the neutral set and 15 of the mutilation set. Each picture was shown two times within the run. Images were not repeated among the runs. The pictures were presented in pseudorandom order, not allowing more than 3 images of the same type to be presented in a roll.

Each run involved the following: a fixation cross presented for 2 to 6 ms, followed by a picture presented for 250 ms. Within a run, there were 60 trials of picture presentation. Additionally, six catch trials occurred randomly along the run. Each run lasted for approximately 4.5 min. For more details on the experimental design, see Bastos et al. [36].

### General linear model analysis

Functional and anatomical data were collected using a 3T scanner (Magnetom Prisma, Siemens, Erlangen, Germany). Imaging data were preprocessed using the Statistical Parametric Mapping (SPM12) software package [40] (Wellcome Department of Imaging Neuroscience, Institute of Neurology, London, UK, https://www.fil.ion.ucl.ac.uk/spm/software/spm12/). For complete details regarding data preprocessing, see the Supplementary Material. For each participant, a regression model was used to determine each voxel coefficient for each condition: neutral pictures in the real context (Real_Neu), mutilation pictures in the real context (Real_Mut), neutral pictures in the safe context (Safe_Neu), mutilation pictures in the safe context (Safe_Mut) and catch trials (CT). The duration of each condition (0 s for neutral and mutilation pictures and 2 s for CT) was convolved with SPM’s canonical haemodynamic response function. Movement parameters were entered into the general linear model (GLM) as covariates.

Four contrast images relative to the baseline were computed for each participant by combining the two runs of each context (safe and real) for each condition (mutilation and neutral). The contrast between mutilation and neutral images during each context was also computed and used as input to the pattern regression analyses. Thus, we used one contrast image per participant for each context analysis (safe and real).

### Pattern regression analysis

We trained two Gaussian process regression (GPR) models [41] to predict PTSD symptoms from brain activation patterns in real and safe contexts. Pattern regression analyses were implemented in the Pattern Recognition for Neuroimaging Toolbox (PRoNTo), version 3 [42]. We obtained similar results using a kernel ridge regression (KRR) model [43]. For brevity, we included only the GPR results in the manuscript (results for the KRR model can be found in the Supplementary Material, Table S2).

**Fig. 1:**
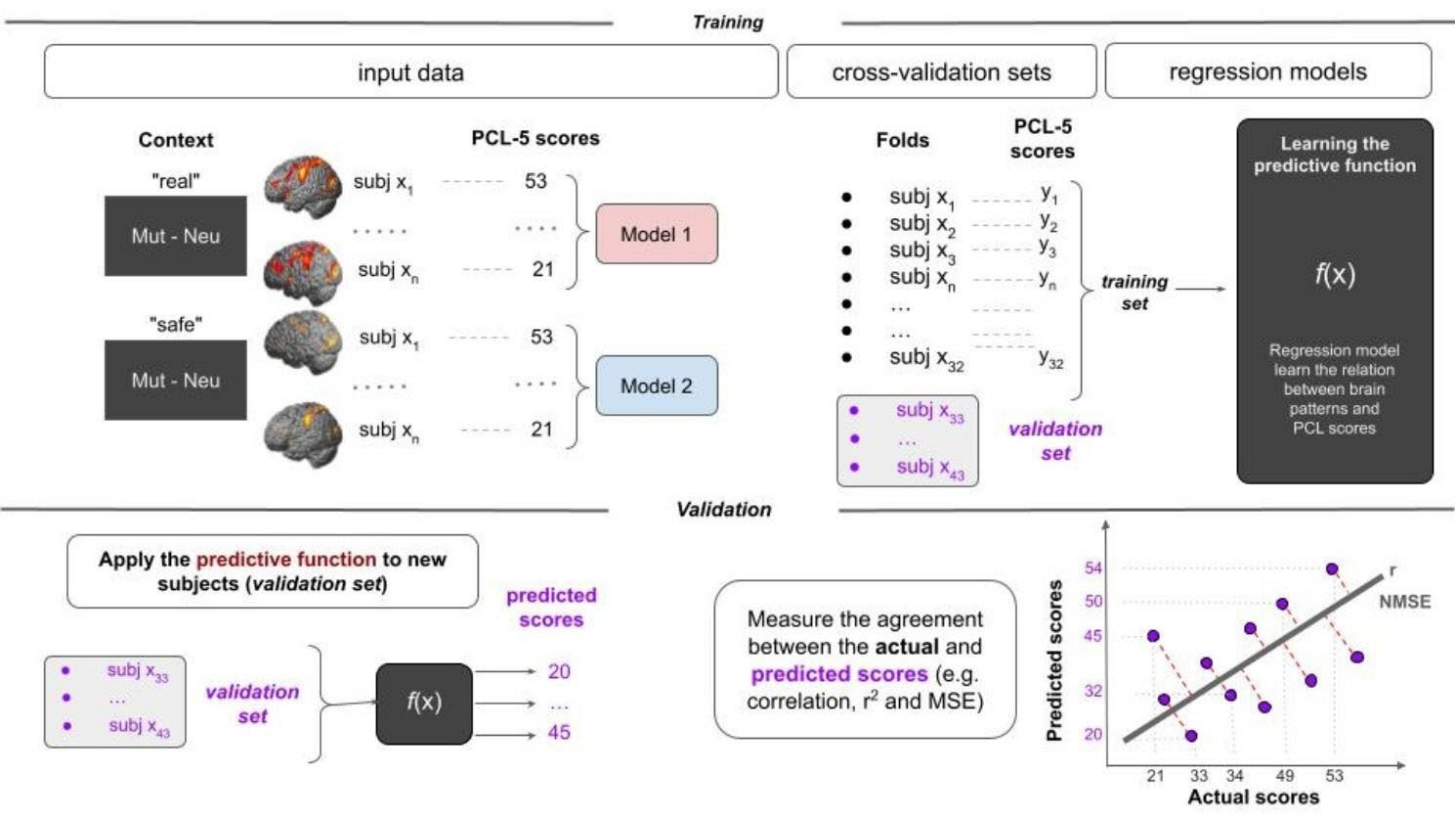
Pattern regression analysis procedure. The training data input for the GPR model consisted of examples that paired the pattern of brain activity elicited by Mut-Neu during the real context (Model 1) and safe context (Model 2) of each subject and their corresponding clinical score (PCL-5 scores). In the training step, we developed a GPR model to predict PCL-5 scores from brain patterns. In the validation step, given the patterns of brain activity of a subject in the validation fold, the GPR model predicted the corresponding clinical score (PCL-5 score). The process was repeated for each of the fold divisions. The results for each of the validation sets were concatenated. Model performance was evaluated using three metrics that measured the agreement between the predicted and actual clinical scores: Pearson’s correlation coefficient (r), the coefficient of determination (R^2^), and the normalized mean squared error (NMSE)

To evaluate the GPR performance, we chose fivefold cross-validation, which involved dividing the data into five separate sets. Four of the sets are used for training the model, and one set is used for testing the model. This procedure is repeated five times, leaving each set out once. Then, the model’s performance is computed by concatenating the predictions on the validation set of all folds. Furthermore, we also used a threefold cross-validation strategy to demonstrate that the results were not dependent on a specific cross-validation scheme (results can be found in the Supplementary Material, Table S2). For the significant model(s), to identify whether it was possible to predict clusters of symptoms of PTSD, we trained four GPR models separately (for intrusion, avoidance, negative alterations in cognition and mood, and arousal/reactivity). For the sake of brevity, we included only the fivefold cross-validation results in the manuscript. A detailed description of the pattern regression analysis procedure is presented in Fig. 1.

#### Model performance

The evaluation of the pattern regression models’ performance was based on three agreement metrics between the predicted and actual scores: the correlation coefficient (r), coefficient of determination (R²), and normalized mean squared error (NMSE). The correlation coefficient (r) quantifies the linear dependence between two variables [44]. In this case, the relationship between predicted and actual PTSD symptoms. A lower value of r indicates poor model predictive performance. The coefficient of determination (R²) indicates the proportion of variance from the original variables explained by the regression model [45, 46]. An R² closer to one indicates a good prediction of the outcome. The mean squared error (MSE) is a standard measure used to assess the goodness-of-fit for regression models [47, 48]. It is computed by the sum of the square difference between the actual and predicted PCL-5 scores (output of the GPR model) for each data point and divided by the sum of the total number of data points. To account for the variance in the target values, the MSE was normalized by dividing by the variance (NMSE). We performed a nonparametric permutation test to assess the significance of the model performance measures. This test involved repeating the cross-validation procedure described above 1,000 times with the labels (the value of PCL-5 scores) permuted across subjects. The test assumes the null hypothesis that the relationship between the data and the labels cannot be reliably learned by the algorithms used in the training step. The alternative hypothesis is that we can train algorithms with small, expected errors. The p-value was calculated by determining the number of times that the absolute value of the metric with the permuted labels was equal to or greater (less for the MSE) than the absolute value of the metric obtained using the correct labels and dividing by 1,000. We employed the Bonferroni correction for multiple comparisons (2 contexts: safe and neutral). Therefore, the results were considered statistically significant if the p-value was less than 0.05/2 = 0.025 [42]. Thus, significant results mean that the model trained in a permuted data with no real association between brain patterns and PCL-5 scores yielded results equal or better than the model trained with the actual data for less than 2,5% of the permuted sets.

#### Covariates

Age, sex, and medication were considered potential covariates that could affect the patterns of brain activity. However, including variables associated with the variable of interest (i.e., the PCL-5) as covariates is not recommended since it will likely remove variability in the associated data [45, 49]. Skewness/Kurtosis tests for normality were carried out to investigate the distribution of the variables. The tests indicated that the age and the PCL-5 scores did not follow a normal distribution (age: 𝛘² = 6.66; p-value = 0.03; PCL-5 scores: 𝛘² = 16.27; p-value < 0.00).

Then, in the present study, we performed a Mann‒Whitney test to determine whether medication and sex were systematically related to PTSD symptoms and a Spearman correlation analysis to determine whether age was associated with PTSD symptoms. The variables not systematically related to the PCL-5 scores were included as covariates in the pattern regression models using an approach that accounted for the training and testing separation [45].

#### Model interpretation

We computed the GPR models’ weight maps for statistically significant r, R², and NMSE values. Weight maps show the relative contribution of each voxel to the predictive function. As previously discussed in the literature [44, 50], the weight maps of machine learning models cannot be thresholded to make regionally specific inferences as in a standard mass-univariate analysis. Since each cross-validation fold yields a different weight vector, the final weight map is an average across the folds’ results. We summarized the weight maps in anatomical regions using an anatomical atlas [16, 17, 44]. We computed the normalized weight for each brain region as the mean of the absolute weights of the voxels within the region. We then ranked the regions according to the percentage of the total normalized weights they explained. We used the Anatomical Automatic Labelling (AAL) atlas [51] from the WFU-PickAtlas [52] toolbox in SPM to define the brain regions (a region-based pattern localization map).

## Results

### Effect of potential confounders on PTSD symptoms

In the present study, medicated participants had significantly different on the PCL-5 scores than unmedicated participants (p-value *=* 0.001; medicated: mean = 49.1, SD = 2.9; unmedicated: mean = 16.7, SD = 19.1). Female participants did not score significantly differently than male participants (p-value = 0.77; female: mean = 26.9, SD = 21.3; male: mean = 23.0, SD.=23.7). There was no significant Spearman correlation between age and PTSD symptoms (rho = 0.20, p-value = 0.18). Thus, we included only age and sex as covariates to regress out these effects from the data. Due to the observed association between PTSD total scores and medication, we could not exclude a potential effect of this covariate on the predictive models. However, to address this limitation, we balanced the proportion of participants with and without medication across folds. There was no difference in the sample distribution regarding medication for the fivefold cross-validation strategy (p-value = 0.43). Furthermore, we balanced the proportion of data to ensure that the distribution of the variable of interest (PCL-5 scores) did not differ significantly among the folds (Kruskal‒Wallis ANOVA, χ² = 2.59, p-value = 0.63).

### Pattern regression model

We used the brain images captured while observing aversive and neutral pictures in safe and real contexts. To predict PTSD symptoms from brain activation patterns while viewing these images, we used two machine learning regression models, including sex and age as covariates.

#### Model 1: real context

**Fig. 2:**
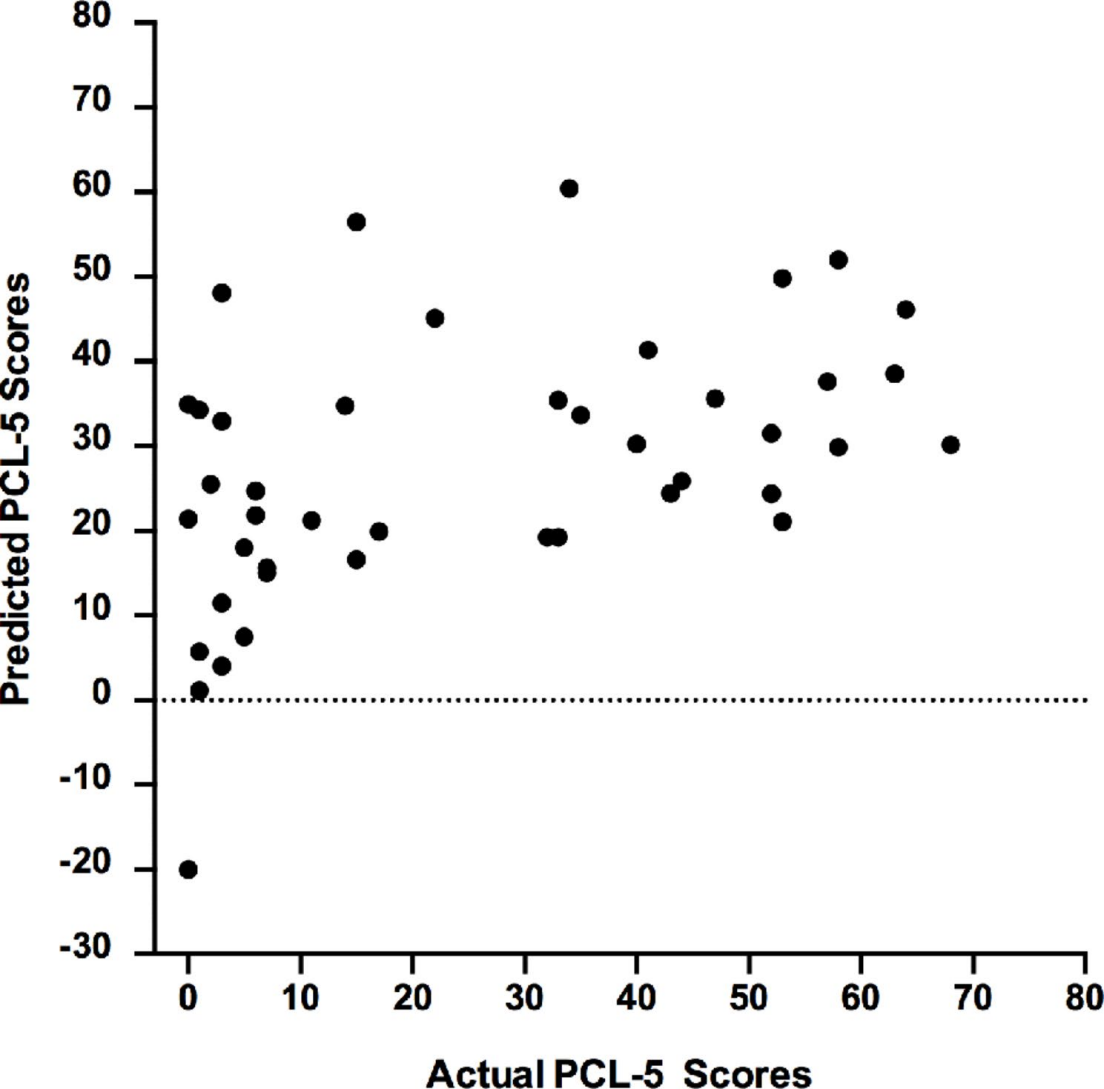
Scatter plot showing actual vs. predicted PTSD scores obtained by the GPR model based on brain activation evoked during the real context using a fivefold cross-validation scheme

The PCL-5 scores estimated by the GPR model from brain activation patterns while viewing mutilation pictures relative to neutral pictures in the real context had a significant correlation with the actual PCL-5 values. After controlling for covariates, r, R² and NMSE between values of the relationship between the estimated and actual PCL-5 were 0.59 (p-value < 0.001), 0.38 (p-value = 0.004), and 0.76 (p-value = 0.004), respectively (Table 1; Fig. 2). The results for each separate fold can be found in Supplementary Materials in Table S3.

**Table 1:** Measurements of agreement between the actual and decoded scores based on the whole-brain activity patterns in response to negative pictures during a real context (Mut-Neu) and a safe context (Mut-Neu)

Models	*Measures of agreement*		
	*r (p-value)*	*R* ^*2*^ *(p-value)*	*NMSE (p-value)*
Real Context (Mut > Neu)	**0.59** ***(0.001)***	**0.38** ***(0.004)***	**0.76** ***(0.004)***
Safe Context (Mut > Neu)	0.01 *(0.53)*	0.02 *(0.99)*	1.41 *(0.85)*

Significance threshold p < 0.05/2 = 0.025. Significant results are displayed in ***bold***

#### Model 2: safe context

The GPR model did not perform better than chance in predicting PTSD symptoms from brain activation patterns while viewing mutilation pictures relative to neutral pictures presented in a safe context. After controlling for covariates, r, R² and NMSE values of the relationship between the decoded and actual PCL-5 were 0.01 (p-value = 0.53), 0.02 (p-value = 0.99), and 1.41 (p-value = 0.85), respectively (Table 1). In summary, the GPR model significantly predicted PTSD symptoms from brain activation patterns when viewing negative pictures in the real context but not in the safe context.

### Contributions of regions to regression model 1 (real context)

**Fig. 3:**
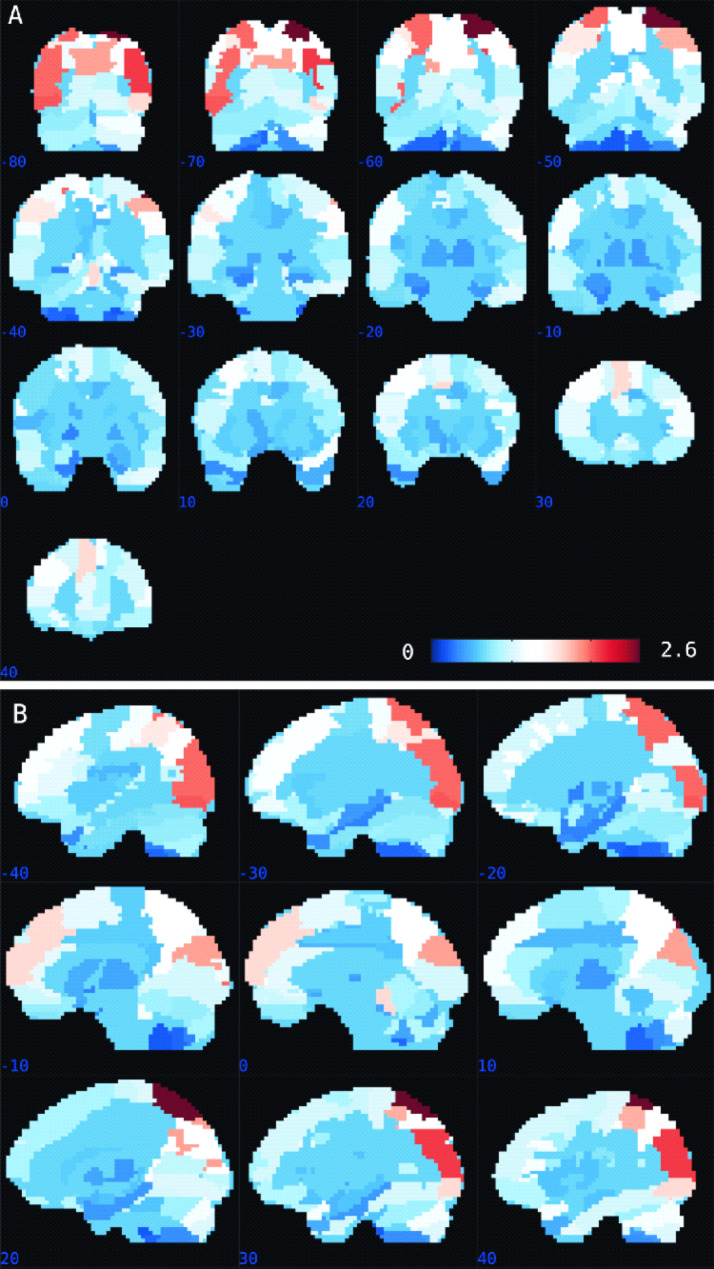
Region-based pattern localization map, a post hoc summarization map computed from the voxel-based predictive pattern. Summary of the contribution of each anatomical region based on the Anatomical Automatic Labelling (AAL) atlas for the GPR model that predicted PCL-5 scores based on patterns of brain activation elicited by negative images (compared to neutral images) during the real context. Panels **(A, B)** represent coronal slices and sagittal slices of the brain, respectively. The color bar indicates the percentage of contribution of each ROI to the model predicting clinical scores

Figure 3 presents the region-based pattern localization map summarizing the brain weight map for the GPR model that predicted PCL-5 scores from the brain activation patterns for mutilation pictures relative to neutral pictures in the real context. The pattern localization map computed from the voxel-based predictive pattern is presented in Table 2 and S4. As mentioned, the pattern localization methodology was based on the AAL atlas [51] to summarize the weight map regarding anatomical regions [16, 17, 44]. Table 2 shows the top 20 ranked regions according to normalized weights per region, representing 31.9% of the total weights of the predictive function (Table S4 shows the relative contribution of all brain regions and can be found in the Supplementary Material). The regions with the highest contributions were the parietal, occipital, and frontal regions. However, it is relevant to mention that the contributions of each region were small.

**Table 2:** Top 20 ranked regions according to normalized weights per region, which represent 31.9% of the total weights of the predictive function

Rank	Anatomical regions	Label	Weight
1	Superior parietal gyrus R	P1	2.60
2	Middle occipital gyrus R	O2	2.07
3	Inferior occipital gyrus L	O3	2.00
4	Superior parietal gyrus L	P1	1.94
5	Middle occipital gyrus L	O2	1.93
6	Cuneus R	Q	1.78
7	Cuneus L	Q	1.77
8	Inferior parietal cortex R	P2	1.73
9	Superior frontal gyrus, medial L	F1M	1.58
10	Inferior occipital gyrus R	O3	1.57
11	Cerebelum vermis III		1.57
12	Inferior parietal cortex L	P2	1.52
13	Supramarginal gyrus R	SMG	1.32
14	Angular gyrus L	AG	1.32
15	Angular gyrus R	AG	1.27
16	Precuneus R	PQ	1.27
17	Superior occipital gyrus R	O1	1.21
18	Precuneus L	PQ	1.20
19	Middle frontal gyrus L	F2	1.16
20	Cerebelum rus IR		1.15

*Abbreviations: R, right; L, left.*^***a***^*Anatomical description based on Automated Anatomical Labelling (AAL) by Tzourio-Mazoyer* et al. *(2002)*

### Models to predict clusters of PTSD symptoms

As previously mentioned, since the GPR model was able to predict total PTSD scores from whole-brain activity patterns in the real context, we ran an additional analysis to predict clusters of symptoms of PTSD in this context only. As might be expected, the PCL subscales scores were highly and significantly correlated with each other (Table S5, Spearman correlation coefficient, rho). We trained four GPR models separately. We employed the Bonferroni correction for multiple comparisons (4 clusters: B, C, D and E), and the results were considered statistically significant if the p-value was less than 0.05/4 = 0.0125 for at least two metrics of correlation. After controlling for covariates and multiple comparisons effects, the GPR model was still able to predict intrusion scores (r = 0.60, p-value = 0.002; R^2^ = 0.38, p-value = 0.006; and NMSE = 0.68, p-value = 0.003) and avoidance scores (r = 0.56, p-value = 0.002; R^2^ = 0.34, p-value = 0.008; and NMSE = 0.86, p-value = 0.01) for the three agreement metrics. Interestingly, the GPR model performed significantly better than chance in predicting alterations in cognition and mood scores in two metrics of agreement (r = 0.56, p-value = 0.003; R^2^ = 0.34, p-value = 0.006), and at borderline for NMSE = 0.80 (p-value = 0.018) after controlling for multiple comparison effects. The GPR regression model did not perform better than chance in predicting the arousal scores. These results are summarized in Table 3. Due to space limitations and to avoid including many tables in the main manuscript, we chose to display tables with the highest contribution to decoding subscale scores for PTSD domains in the supplemental material (Tables S6, S7 and S8). As expected, this analysis yielded very similar regions to those obtained in Model 1 (the GPR model that predicted PTSD total symptoms from the brain activation patterns for mutilation pictures relative to neutral pictures in the real context), including the parietal, occipital, and frontal regions.

**Table 3:** Measurements of agreement between the actual and decoded subscale scores based on the whole-brain activity patterns in response to viewing negative pictures in areal context (Mut-Neu)

Models	*Measures of agreement*		
	*r (p value)*	*R* ^*2*^ *(p-value)*	*NMSE (p value)*
Intrusion (Cluster B)	**0.60** ***(0.002)***	**0.38** ***(0.006)***	**0.68** ***(0.003)***
Avoidance (Cluster C)	**0.56** ***(0.002)***	**0.34** ***(0.008)***	**0.86** ***(0.01)***
Cognition and Mood (Cluster D)	**0.56** ***(0.003)***	**0.37** ***(0.006)***	0.80 *(0.018****)***
Arousal (Cluster E)	**0.51** ***(0.005)***	0.29 (0.02)	0.87 (0.06)

Statistically significant p < 0.05/4 = 0.0125. Significant results are displayed in ***bold***

## Discussion

The current study indicated that the GPR model could capture the association between PTSD total scores and the whole-brain activity patterns elicited by mutilation pictures (compared to neutral pictures) in the real context but not in the safe context. Additional analysis showed that the GPR model-estimated values for 3 of 4 clusters of PTSD symptoms (intrusion, avoidance and cognition and mood) which were significantly correlated with the actual values. The clusters for intrusion and avoidance achieved statistical significance for all three-agreement metrics used after the Bonferroni correction. The brain regions with the highest contribution to the model were the occipito-parietal regions, including the superior parietal gyrus, inferior parietal gyrus, and supramarginal gyrus. Our results indicated that this pattern of brain activity could potentially be a biomarker for PTSD symptoms. Nevertheless, contrary to our expectations, the GPR model estimates for PCL-5 scores were not significantly correlated with the actual values in the safe context.

Neuroimaging studies that applied pattern regression to retrieve associations to PTSD symptoms at the individual level are scarce and have mainly utilized rs-fMRI data [22, 24, 25, 53]. To date, this is the first study to apply pattern regression analysis to find brain signatures while participants perform specific tasks during an fMRI scan. In the present study, the participants were exposed to human mutilation pictures and equivalent neutral pictures in two different contexts: “real” and “safe”. We employed this category of pictures since it is well known that viewing an injured individual (as present in mutilation pictures) may signal a potential life threat in the environment and could induce defensive responses in humans [54, 55]. Furthermore, evidence from fMRI [56, 57] showed that images of mutilated bodies compared to neutral ones activated classically emotional areas such as the amygdala, insula, and midcingulate cortex [58].

Here, the GPR model could retrieve the association between PTSD total scores and whole-brain activity patterns elicited by mutilation pictures (compared to neutral pictures) in the real context. Consistent with our present findings, previous studies by our group have shown an association between EEG data elicited by mutilation pictures in the real context and PTSD symptoms in a trauma-exposed cohort [30, 59]. Taken together, our results corroborate the evidence that the pattern of brain activity involved in threat processing and implementation of defensive responses in a highly aversive context might represent a neurobiological marker for PTSD symptoms [30, 31, 60, 61].

Regarding the prediction of PTSD subscale scores, the results should be interpreted with caution, as the PCL-5 subscales are highly correlated with each other (Supplemental Material). However, as the subscales measure different aspects of PTSD pathology, some clusters could be more strongly associated with the task than others. The present results indicated that GPR models were able to significantly decode PTSD symptoms in the clusters of intrusion (cluster B), avoidance (cluster C), and alteration in mood and cognition (cluster D) from patterns of brain activity elicited by mutilation pictures (compared to neutral pictures) in the real context. Although the results are promising, we acknowledge that the model estimates for arousal symptoms were not achieve a significance level in the agreement metrics. One possible explanation is due to our modest sample size or differences in symptom representation. To date, only one other study has applied pattern regression models to clusters of PTSD symptoms [24]. The study was unable to predict cognition and arousal scores but linked severity of different PTSD symptom clusters to distinct patterns of network connectivity [24]. In contrast to Zandvakili et al. [24], our results were highly similar across different clusters of symptoms, and the observed differences in arousal may be attributed to a relatively small sample size and power issues. Further research in larger samples is needed to properly address this issue.

However, contrary to our expectations, the GPR model estimates of PCL-5 scores were not significantly correlated with the actual scores in the safe context. We adopted this experimental approach in the present study since it is well established that the inability to engage in safety cues is a relevant biomarker for PTSD [27–29]. Many factors might explain this unexpected result. One possibility is that the valence effect in this experimental paradigm is highly variable in patients with PTSD [36], possibly due to the different types and amounts of trauma in our sample. In the safe context, the variable engagement in safety cues may have added another layer of variability, which further reduced the ability of our model to estimate PTSD scores from brain activity. This finding aligns with those of other studies showing that more traumatized individuals have different reactivity patterns to aversive cues [62] and possibly engage differently in safety cues. This heterogeneity may represent substantial variability in activated brain regions, thereby preventing the development of an efficient model capable of retrieving the association between PTSD symptoms and brain patterns, especially in the safe context, with this sample size. In summary, in the real context, the pattern of brain activation may be more homogeneous among trauma-exposed participants, as reactions to very aversive stimuli can generate more consistent network activation. Although mutilation is not a trauma-relevant image for all traumatized subjects, it elicits intense emotional reactions in the general population [54, 63]. On the other hand, in the safe context, these networks of brain regions may be more diverse among trauma participants, preventing the GPR models from identifying a pattern to differentiate this group.

The brain regions with the highest contributions to the model obtained for activity elicited by viewing mutilation pictures in the real context were occipitoparietal regions, including the superior parietal gyrus (BA7), inferior parietal gyrus (IPG, BA40), and supramarginal gyrus. A recent review focusing on fMRI affective processing paradigms provided partial support for the traditional functional neurocircuitry mode (FNM) of PTSD, in which individuals with PTSD exhibit hyperactivation of the amygdala, contributing to heightened processing of negative stimuli, and hypoactivation of the medial prefrontal cortex, resulting in inappropriate and persistent fear of trauma- and nontrauma-related stimuli [26]. However, the authors argued that the FNM model might underrepresent the neurobiological complexity of PTSD. In this context, our work contributes to the literature by highlighting the importance of the occipitoparietal regions for affective processing paradigms. For example, the visual cortex has been viewed as part of a neurocircuit that regulates stress responses to emotional images [64]. A previous study showed atrophy of the visual cortex in children and adolescents with reactive attachment disorders. The authors argued that these visual cortex abnormalities may also be associated with impairments in visual emotion regulation, leading to an increased risk of later psychopathology [65]. In addition, in humans, the superior parietal gyrus, inferior parietal gyrus, and supramarginal gyrus are all part of the posterior parietal lobe, which integrates visuospatial and somatosensory information to shape an appropriate motor response [66]. Specifically, the superior parietal cortex and other integrative regions may monitor, predict, and evade intrusive actions toward the body [67, 68]. Our findings align with those of our previous study [36] that showed enhanced BOLD activity in the supramarginal gyrus when participants viewed mutilation pictures compared to neutral pictures in the real context. Furthermore, parietal regions are involved in the processing of mutilation pictures [56, 57, 69] as well as the coordination of defensive responses [70]. Thus, changes in activity in these regions when processing mutilated pictures could be related to changes in visuospatial and somatosensory responses related to preparation for defense. As a result, these findings suggest that alterations in regions important for defensive reactions may serve as a biomarker for PTSD symptoms at the individual level. Considering the brain regions with the highest contributions to the model estimating PTSD symptom clusters, as expected, we obtained very similar regions as those obtained with Model 1, i.e., the GPR model for retrieving the association of PTSD total symptoms from the brain activation patterns elicited by mutilation pictures relative to neutral pictures in the real context, including the parietal, occipital, and frontal regions. However, the contributions of individual regions to the predictive model were small, indicating that the model was based on the overall pattern rather than on a small combination of regions. Schrouff and Mourao-Miranda [50] have shown that when brain pattern differences differ subtly among the compared groups, the weights are more distributed across all brain regions.

## Limitations

The main limitation was that the medication variable was associated with the variable that we wanted to predict (PCL-5 scores). Therefore, removing their effect from patterns of brain activity would also remove the variability in the data associated with the clinical scores. Additionally, the results might not be generalizable to other samples due to the relatively small sample size. Finally, the GPR models yielded predictive patterns that were difficult to interpret regarding the underlying neurobiology. The models’ weights were distributed across the whole brain, and the evidence of contributions of specific brain regions to the predictions should be carefully interpreted.

## Conclusion

The present study aimed to combine fMRI and pattern regression analysis to identify patterns of brain activity elicited by mutilation pictures relative to neutral pictures to predict PTSD symptoms in two contexts: a real context and a safe context. Our results showed that it was possible to predict PTSD symptoms from patterns of brain activity elicited by mutilation pictures in the real context. Additional analysis showed that GPR models accurately predicted 3 of 4 clusters of PTSD symptoms: the intrusion, avoidance and alteration in cognition and mood clusters. Nevertheless, contrary to our expectations, the model could not decode PTSD symptoms in the safe context. In summary, our results demonstrated that mutilation pictures, which have higher levels of negative emotional content, could be useful in identifying potential biomarkers for PTSD symptoms, especially in occipitoparietal regions. Our results support the RDoC recommendation of searching for biomarkers in a sample spanning the full range of symptoms, from normal to abnormal. Further studies combining fMRI data and machine learning approaches can contribute to the search for potential biomarkers reflecting mechanisms of mental disorders in transdiagnostic samples.

### Electronic supplementary material

Below is the link to the electronic supplementary material.

Supplementary Material 1: Portugal_2023

## Data Availability

Data will be made available on request. The datasets generated and analyzed during the current study are not publicly available because we did not seek approval from the ethics committee to make the data accessible to the general public. However, interested parties can obtain the data from the corresponding author upon reasonable request.

## Abbreviations

PTSD: Posttraumatic stress disorder
RDoC: Research Domain Criteria
fMRI: Functional magnetic resonance imaging
PCL-5: Posttraumatic Stress Disorder Checklist for the DSM-5
Real_Neu: Neutral pictures in the real context
Real_Mut: Mutilation pictures in the real context
Safe_Neu: Neutral pictures in the safe context
Safe_Mut: Mutilation pictures in the safe context
GPR: Gaussian process regression
r: Correlation coefficient
r²: Coefficient of determination
NMSE: Normalized mean squared error
