# Supplementary material for: Machine learning applied to fMRI patterns of brain activation in response to mutilation pictures predicts PTSD symptoms
Source: BMC Psychiatry. 2023 Oct 5;23:719. doi: 10.1186/s12888-023-05220-x (PMC10552290; doi:10.1186/s12888-023-05220-x)
Supplement: Supplementary file 1 — Supplementary Material 1: Portugal_2023 [file 12888_2023_5220_MOESM1_ESM.docx]

**Supplemental Material**

***Posttraumatic Stress Disorder Checklist for the DSM-5***

This scale was translated and adapted to Portuguese by Lima et al. (2016). The PCL-5 is a 20-item self-report questionnaire that measures four clusters of symptoms of PTSD: intrusion, avoidance, negative alterations in cognition and mood, and alterations in arousal and reactivity. Each item on the PCL-5 is scored on a five-point Likert scale (from 0 = “not at all” to 4 = “extremely”). Symptom severity can be calculated by summing the item scores for each of the four clusters or summing all 20 items: the total severity score ranges from zero to 80 points.

***Data Preprocessing***

Functional and anatomical data were collected using a 3T scanner (Magnetom Prisma, Siemens, Erlangen, Germany) from 23 patients with PTSD and 29 trauma-exposed participants. Functional data were visually inspected for quality and movement artifacts using the ArtRepair toolbox (v3, 2009) (Mazaika et al., 2009, 2007). In this step, no subjects were removed. Imaging data were preprocessed and analyzed using the Statistical Parametric Mapping (SPM12) software package (Wellcome Department of Imaging Neuroscience, Institute of Neurology, London, UK; http://www.fil.ion.ucl.ac.uk/spm12/). Images were slice time corrected and realigned using the first functional volume of each run as a reference, coregistered with the participant's magnetization-prepared rapid acquisition gradient echo (MP-RAGE) image, segmented, normalized into standard stereotactic space (Montreal Neurologic Institute, MNI; http://www.bic.mni.mcgill.ca), and spatially smoothed using a Gaussian kernel (full width at half maximum (FWHM): 8 mm). After the preprocessing procedure, we used ArtRepair software to compute the frame-to-frame movement on the volumes, and subjects showing more than 20% of images exceeding the threshold of 0.5 mm/TR (Mazaika, 2015) were excluded from the analysis. The volumes were realigned using a six-parameter rigid body transformation, and those parameters were later regressed out from the realigned data. In total, eight participants were excluded due to excessive movement (three from the PTSD group and five controls). One control participant was excluded for presenting a subclinical score for a PTSD diagnosis. The final sample included 43 subjects, 20 PTSD patients, and 23 controls.

**Table S1:** Clinical and sociodemographic characteristics of participants.

| **Variable** | **Sample (*n* = 43)** |
| --- | --- |
| **Sex (n)** | 30 females, 13 males |
| **Age, years (m ± SD)** | 31.5 ± 12.3 |
| **PCL-5 score (mean; min - max)** | 25.7 (0-68) |
| **Cluster B score (intrusion symptoms)** | 6.4 (0-16) |
| **Cluster C score (persistent avoidance)** | 3.5 (0-8) |
| **Cluster D score (negative alterations in cognitions and mood)** | 8.3 (0-28) |
| **Cluster E score (alterations in arousal and reactivity)** | 7.5 (0-20) |
| **Trauma type (LEC-5 score) (n):** |  |
| **Assault with a weapon** | 10 |
| **Sudden accidental death** | 5 |
| **Sexual assault** | 7 |
| **Captivity** | 6 |
| **Physical assault** | 4 |
| **Transportation accident** | 2 |
| **Life-threatening illness or injury** | 2 |
| **Natural disaster** | 2 |
| **Fire or explosion** | 1 |
| **Severe human suffering** | 1 |
| **Serious injury, harm, or death you caused to someone else** | 1 |
| **Any other very stressful event or experience** | 2 |
| **Psychiatric medication used (n):** |  |
| **Citalopram** | 1 |
| **Escitalopram** | 10 |
| **Paroxetine** | 1 |

**Table S2:** Measurements of agreement between the actual and estimated scores based on whole-brain activity patterns in response to negative images during the real context (Mut-Neu) and safe context (Mut-Neu).

| ***Model*** | ***Algorithm*** | ***Folds*** | ***Measures of agreement*** | | |
| --- | --- | --- | --- | --- | --- |
|  |  |  | ***r (p value)*** | ***R^2^ (p value)*** | ***NMSE (p value)*** |
| Real context  (Mut-Neu) | GPR | Three | 0.43 (0.01) | 0.19 (0.04) | 0.86 (0.04) |
|  | KRR | Five | 0.59 (0.002) | 0.38 (0.006) | 0.76 (0.01) |
|  |  | Three | 0.43 (0.01) | 0.19 (0.05) | 0.87 (0.03) |
| Safe context  (Mut-Neu) | GPR | Three | 0.16 (0.22) | 0.04 (0.70) | 1.31 (0.70) |
|  | KRR | Five | 0.01 (0.46) | 0.02 (0.99) | 1.39 (0.83) |
|  |  | Three | 0.16 (0.21) | 0.04 (0.71) | 1.30 (0.68) |

**Table S3:** Measurements of agreement between the actual and estimated scores based on whole-brain activity patterns in response to negative images during the real context (Mut-Neu) for each separate fold.

| ***Model*** | ***Folds*** | ***Measures of agreement*** | | |
| --- | --- | --- | --- | --- |
|  |  | ***r*** | ***R^2^*** | ***NMSE*** |
| Real context  (Mut-Neu) GPR | One | 0.46 | 0.21 | 1.25 |
|  | Two | 0.43 | 0.19 | 0.94 |
|  | Three | 0.83 | 0.69 | 0.41 |
|  | Four | 0.83 | 0.69 | 0.37 |
|  | Five | 0.39 | 0.15 | 0.84 |

**Table S4:** All regions according to normalized weights per region, which represent total weights of the predictive function.

| **Rank** | **Label** | **Weight** |
| --- | --- | --- |
| 1 | Parietal_Sup_R | 2.60 |
| 2 | Occipital_Mid_R | 2.07 |
| 3 | Occipital_Inf_L | 2.00 |
| 4 | Parietal_Sup_L | 1.94 |
| 5 | Occipital_Mid_L | 1.93 |
| 6 | Cuneus_R | 1.78 |
| 7 | Cuneus_L | 1.77 |
| 8 | Parietal_Inf_R | 1.73 |
| 9 | Frontal_Sup_Medial_L | 1.58 |
| 10 | Occipital_Inf_R | 1.57 |
| 11 | Vermis_3 | 1.57 |
| 12 | Parietal_Inf_L | 1.52 |
| 13 | SupraMarginal_R | 1.32 |
| 14 | Angular_L | 1.32 |
| 15 | Angular_R | 1.27 |
| 16 | Precuneus_R | 1.27 |
| 17 | Occipital_Sup_R | 1.21 |
| 18 | Precuneus_L | 1.20 |
| 19 | Frontal_Mid_L | 1.16 |
| 20 | Cerebellum_Crus1_R | 1.15 |
| 21 | SupraMarginal_L | 1.15 |
| 22 | Frontal_Inf_Tri_R | 1.14 |
| 23 | Frontal_Sup_Medial_R | 1.14 |
| 24 | Temporal_Pole_Sup_R | 1.13 |
| 25 | Postcentral_L | 1.13 |
| 26 | Cerebellum_3_L | 1.12 |
| 27 | Occipital_Sup_L | 1.10 |
| 28 | Frontal_Mid_Orb_L | 1.09 |
| 29 | Cingulum_Ant_L | 1.06 |
| 30 | Frontal_Inf_Tri_L | 1.06 |
| 31 | Temporal_Inf_R | 1.05 |
| 32 | Supp_Motor_Area_L | 1.04 |
| 33 | Postcentral_R | 1.03 |
| 34 | Lingual_R | 1.03 |
| 35 | Cerebellum_Crus2_R | 1.00 |
| 36 | Frontal_Sup_L | 0.99 |
| 37 | Fusiform_R | 0.99 |
| 38 | Frontal_Mid_R | 0.98 |
| 39 | Calcarine_L | 0.98 |
| 40 | Frontal_Mid_Orb_R | 0.98 |
| 41 | Lingual_L | 0.96 |
| 42 | Frontal_Inf_Oper_L | 0.95 |
| 43 | Temporal_Mid_L | 0.95 |
| 44 | Frontal_Mid_Orb_L | 0.93 |
| 45 | Precentral_R | 0.92 |
| 46 | Cerebellum_3_R | 0.91 |
| 47 | Temporal_Mid_R | 0.90 |
| 48 | Temporal_Sup_L | 0.88 |
| 49 | Calcarine_R | 0.87 |
| 50 | Frontal_Mid_Orb_R | 0.87 |
| 51 | Frontal_Inf_Orb_R | 0.85 |
| 52 | Vermis_10 | 0.85 |
| 53 | Vermis_8 | 0.84 |
| 54 | Paracentral_Lobule_R | 0.83 |
| 55 | Cingulum_Ant_R | 0.83 |
| 56 | Amygdala_L | 0.82 |
| 57 | Cerebellum_Crus2_L | 0.81 |
| 58 | Frontal_Sup_R | 0.81 |
| 59 | Frontal_Inf_Orb_L | 0.80 |
| 60 | Vermis_4_5 | 0.80 |
| 61 | Frontal_Inf_Oper_R | 0.79 |
| 62 | Cerebellum_6_R | 0.79 |
| 63 | Temporal_Sup_R | 0.78 |
| 64 | Temporal_Pole_Sup_L | 0.78 |
| 65 | Cerebellum_4_5_L | 0.76 |
| 66 | Cerebellum_Crus1_L | 0.75 |
| 67 | Cingulum_Post_R | 0.74 |
| 68 | Supp_Motor_Area_R | 0.73 |
| 69 | Rectus_L | 0.73 |
| 70 | Vermis_6 | 0.72 |
| 71 | Cerebellum_6_L | 0.71 |
| 72 | Fusiform_L | 0.69 |
| 73 | Rolandic_Oper_R | 0.69 |
| 74 | Frontal_Sup_Orb_L | 0.68 |
| 75 | Rectus_R | 0.64 |
| 76 | Precentral_L | 0.64 |
| 77 | Temporal_Inf_L | 0.63 |
| 78 | Frontal_Sup_Orb_R | 0.62 |
| 79 | Putamen_R | 0.62 |
| 80 | Putamen_L | 0.61 |
| 81 | others | 0.60 |
| 82 | Heschl_L | 0.59 |
| 83 | Heschl_R | 0.59 |
| 84 | Insula_L | 0.57 |
| 85 | Paracentral_Lobule_L | 0.56 |
| 86 | Cingulum_Mid_L | 0.56 |
| 87 | Caudate_R | 0.55 |
| 88 | Cingulum_Post_L | 0.52 |
| 89 | Cerebellum_4_5_R | 0.52 |
| 90 | Insula_R | 0.51 |
| 91 | Hippocampus_R | 0.51 |
| 92 | Rolandic_Oper_L | 0.51 |
| 93 | Cingulum_Mid_R | 0.50 |
| 94 | Amygdala_R | 0.49 |
| 95 | Olfactory_R | 0.48 |
| 96 | Caudate_L | 0.46 |
| 97 | Temporal_Pole_Mid_R | 0.46 |
| 98 | Vermis_7 | 0.45 |
| 99 | Olfactory_L | 0.43 |
| 100 | Pallidum_R | 0.40 |
| 101 | Vermis_9 | 0.39 |
| 102 | Vermis_1_2 | 0.39 |
| 103 | Thalamus_L | 0.39 |
| 104 | ParaHippocampal_R | 0.38 |
| 105 | Cerebellum_7b_R | 0.38 |
| 106 | Thalamus_R | 0.38 |
| 107 | Hippocampus_L | 0.38 |
| 108 | Temporal_Pole_Mid_L | 0.33 |
| 109 | Cerebellum_8_R | 0.32 |
| 110 | Pallidum_L | 0.29 |
| 111 | Cerebellum_7b_L | 0.29 |
| 112 | ParaHippocampal_L | 0.28 |
| 113 | Cerebellum_9_R | 0.22 |
| 114 | Cerebellum_8_L | 0.21 |
| 115 | Cerebellum_10_L | 0.21 |
| 116 | Cerebellum_9_L | 0.16 |
| 117 | Cerebellum_10_R | 0.14 |

**Table S5:** Correlation matrix of scores on the PCL-5 subscales.

|  | **PCL-5: B** | **PCL-5: C** | **PCL-5: D** | **PCL-5: E** | **PCL-5: total** |
| --- | --- | --- | --- | --- | --- |
| PCL-5: B | 1 | 0.82* | 0.89* | 0.88* | 0.96* |
| PCL-5: C | 0.82* | 1 | 0.86* | 0.71* | 0.86* |
| PCL-5: D | 0.89* | 0.86* | 1 | 0.86* | 0.96* |
| PCL-5: E | 0.88* | 0.71* | 0.86* | 1 | 0.93* |
| PCL-5: total | 0.96* | 0.86* | 0.96* | 0.93* | 1 |

Clusters B, C, D and E correspond to intrusion, persistent avoidance, negative alterations in cognition and mood, and alterations in arousal and reactivity, respectively. The values in the table are Spearman's correlation coefficients (rho). *p < 0.001. The Shapiro‒Wilk test for multivariate normality indicated that all subscales presented a nonparametric distribution (W = 0.868, p = 0.001).

**Table S6:** The top 20 ranked regions according to normalized weights per region, representing 33.3% of the total weights of the predictive function for Cluster B scores.

| **Rank** | **Anatomical region** | **Label** | **Weight** |
| --- | --- | --- | --- |
| 1 | Superior parietal gyrus R | P1 | 2.80 |
| 2 | Inferior occipital gyrus L | O3 | 2.40 |
| 3 | Cuneus R | Q | 2.02 |
| 4 | Middle occipital gyrus R | O2 | 2.00 |
| 5 | Cuneus L | Q | 1.89 |
| 6 | Inferior occipital gyrus R | O3 | 1.81 |
| 7 | Superior parietal gyrus L | P1 | 1.79 |
| 8 | Middle occipital gyrus L | O2 | 1.77 |
| 9 | Cerebellum vermis III | - | 1.70 |
| 10 | Superior frontal gyrus, medial L | F1M | 1.66 |
| 11 | Inferior parietal cortex R | P2 | 1.64 |
| 12 | Inferior parietal cortex L | P2 | 1.63 |
| 13 | SupraMarginal_R | SMG | 1.37 |
| 14 | Angular_R | AG | 1.37 |
| 15 | Cerebellum_3_L | - | 1.30 |
| 16 | Precuneus R | PQ | 1.28 |
| 17 | Postcentral gyrus L | POST | 1.26 |
| 18 | Superior Frontal Gyrus, Medial R | F1M | 1.25 |
| 19 | Supplementary Motor Area L | SMA | 1.20 |
| 20 | Cerebellum_Crus1_R | - | 1.19 |

**Table S7:** The top 20 ranked regions according to normalized weights per region, representing 32.21% of the total weights of the predictive function for Cluster C scores.

| **Rank** | **Anatomical region** | **Label** | **Weight** |
| --- | --- | --- | --- |
| 1 | Superior parietal gyrus R | P1 | 2.90 |
| 2 | Inferior parietal cortex R | P2 | 2.20 |
| 3 | Cuneus R | Q | 2.15 |
| 4 | Middle occipital gyrus R | O2 | 2.05 |
| 5 | Superior parietal gyrus L | P1 | 1.82 |
| 6 | Cuneus L | Q | 1.82 |
| 7 | Cerebellum vermis III | - | 1.69 |
| 8 | Middle occipital gyrus L | O2 | 1.64 |
| 9 | Angular gyrus L | AG | 1.60 |
| 10 | Inferior occipital gyrus L | O3 | 1.46 |
| 11 | Inferior parietal cortex L | P2 | 1.43 |
| 12 | Inferior occipital gyrus R | O3 | 1.41 |
| 13 | Angular gyrus L | AG | 1.40 |
| 14 | Anterior cingulate_L | ACIN | 1.37 |
| 15 | Cerebellum_3_L | - | 1.27 |
| 16 | Superior Frontal Gyrus, medial L | F1M | 1.24 |
| 17 | Inferior frontal gyrus, opercularis part R | F3OP | 1.20 |
| 18 | Superior temporal gyrus_R | T1 | 1.20 |
| 19 | Superior occipital gyrus_R | O1 | 1.19 |
| 20 | Middle frontal gyrus, orbital part_R | F2O | 1.17 |

**Table S8:** The top 20 ranked regions according to normalized weights per region, representing 32.2% of the total weights of the predictive function for Cluster D scores.

| **Rank** | **Anatomical region** | **Label** | **Weight** |
| --- | --- | --- | --- |
| 1 | Superior parietal gyrus R | P1 | 2.64 |
| 2 | Middle occipital gyrus R | O2 | 2.05 |
| 3 | Superior parietal gyrus L | P1 | 2.03 |
| 4 | Middle occipital gyrus L | O2 | 1.95 |
| 5 | Inferior parietal cortex R | P2 | 1.87 |
| 6 | Inferior occipital gyrus L | O3 | 1.87 |
| 7 | Cuneus_L | Q | 1.74 |
| 8 | Cuneus_R | Q | 1.68 |
| 9 | Inferior occipital gyrus R | O3 | 1.59 |
| 10 | Vermis_3 | - | 1.56 |
| 11 | Superior Frontal Gyrus, medial L | F1M | 1.51 |
| 12 | Angular gyrus L | AG | 1.48 |
| 13 | Parietal_Inf_L |  | 1.43 |
| 14 | Anterior cingulate_L | ACIN | 1.35 |
| 15 | Supra Marginal gyrus_R | SMG | 1.29 |
| 16 | Precuneus L | PQ | 1.27 |
| 17 | Precuneus R | PQ | 1.23 |
| 18 | Angular gyrus R | AG | 1.23 |
| 19 | Occipital Superior Gyrus _R | O1 | 1.23 |
| 20 | Inferior frontal gyrus, triangulars part R | F3T | 1.20 |

***Investigating the effect of movement on the results***

To investigate whether the estimated PCL-5 scores were correlated with the amount of movement of each subject, we calculated the root mean square error (RMSE) values for all individual image volumes based on the six movement parameters. Then, we obtained the median RMSE to serve as the subject movement measure (modified from [1,2)](https://www.zotero.org/google-docs/?i81LKN). We computed the Spearman correlation coefficient of this measure with actual PCL-5 scores and the predicted scores (from the GPR model with 5-fold cross-validation in the real context), as well as the difference between them. There was no correlation between movement measures and actual PCL-5 scores (rho = 0.171, *p* = 0.274), predicted PCL-5 scores (rho = 0.042, *p* = 0.791), or the difference between actual and predicted PCL-5 scores (rho = 0.105, *p* = 0.500). Since the measure capturing overall movement was not correlated with the targets/labels, it is unlikely that the GPR findings were due to movement artifacts. In addition, the PTSD group had a mean value of 0.302 mm/TR (SD = 0.183), and the control group had a mean value of 0.262 mm/TR (SD = 0.165). There was no significant variation in head motion between the groups (Kruskal‒Wallis ANOVA, χ² = 0.607, *p* = 0.436). The detailed results are presented in Table S9.

**Table S9:** Descriptive results of movement measures (in mm/TR) for PTSD and control groups and correlation with the predicted PCL-5 scores of the fivefold GPR model in real context:

| ***Group*** | ***N*** | ***Mean*** | ***Std. deviation*** | ***(Min - max)*** | | **95% Confidence interval** | | ***Kruskal‒Wallis Test*** | | |
| --- | --- | --- | --- | --- | --- | --- | --- | --- | --- | --- |
|  |  |  |  |  |  | (Lower - Upper) | | χ² | df | *P value* |
| PTSD | 20 | 0.302 | 0.183 | 0.086 | 0.651 | 0.222 | 0.329 | 0.607 | 1 | 0.436 |
| Control | 23 | 0.262 | 0.165 | 0.081 | 0.643 | 0.194 | 0.329 |  |  |  |
| ***Movement measure correlations:*** | | | | | | | ***Spearman's rho*** | | | *P value* |
| *Actual PCL-5 score* | | | | | | | 0.171 | | | 0.274 |
| *Predicted PCL-5 score* | | | | | | | 0.042 | | | 0.791 |
| *Difference between actual and predicted PCL-5 scores* | | | | | | | 0.105 | | | 0.500 |

Shapiro‒Wilk test for multivariate normality W = 0.884, *p* < 0.001.

References:

1. Lima EDP, Vasconcelos AG, Berger W, Kristensen CH, Nascimento ED, Figueira I, et al. Cross-cultural adaptation of the Posttraumatic Stress Disorder Checklist 5 (PCL-5) and Life Events Checklist 5 (LEC-5) for the Brazilian context. Trends Psychiatry Psychother. 2016;38:207–15

[2.Todd N, Josephs O, Callaghan MF, Lutti A, Weiskopf N. Prospective motion correction of 3D echo-planar imaging data for functional MRI using optical tracking. NeuroImage. 2015 Jun;113:1–12.](https://www.zotero.org/google-docs/?yjS9Oy)

[3.Maziero D, Rondinoni C, Marins T, Stenger VA, Ernst T. Prospective motion correction of fMRI: Improving the quality of resting state data affected by large head motion. NeuroImage. 2020 May;212:116594.](https://www.zotero.org/google-docs/?yjS9Oy)
